# Pregnancy after Treatment for Cervical Cancer Precursor Lesions in a Retrospective Matched Cohort

**DOI:** 10.1371/journal.pone.0117525

**Published:** 2015-02-11

**Authors:** Allison L. Naleway, Sheila Weinmann, Girishanthy Krishnarajah, Bhakti Arondekar, Jovelle Fernandez, Geeta Swamy, Evan Myers

**Affiliations:** 1 The Center for Health Research, Kaiser Permanente Northwest, Portland, OR, United States of America; 2 GlaxoSmithKline, North American Vaccine Development, Philadelphia and King of Prussia, PA, United States of America; 3 Duke University Medical Center, Department of Obstetrics and Gynecology, Durham, NC, United States of America; State University of Maringá/Universidade Estadual de Maringá, BRAZIL

## Abstract

**Objective:**

To determine whether treatments for precancerous cervical lesions were associated with lower pregnancy rates compared to rates in unexposed women and women who had a diagnostic cervical biopsy or colposcopy.

**Design:**

Matched, retrospective cohort study.

**Setting:**

Kaiser Permanente Northwest (KPNW), an integrated healthcare delivery system in Oregon and Washington.

**Patients:**

Women 14 to 53 years old with KPNW enrollment during the period 1998 through 2009.

**Main Outcome Measure:**

Pregnancy after exposure or index date. Pregnancy was defined using a validated algorithm and electronic medical records data.

**Results:**

We observed 570 pregnancies following cervical treatment in 4,137 women, 1,533 pregnancies following a diagnostic procedure in 13,767 women, and 7,436 pregnancies in a frequency-matched sample of 81,435 women unexposed to treatment or diagnostic procedures. After adjusting for age and contraceptive use, we observed a higher rate of pregnancies in the treatment group compared to unexposed women (hazard ratio (HR) = 1.42, 95% confidence interval (CI): 1.30–1.55), but no difference in pregnancy rates between the treatment and diagnostic procedure groups (HR = 1.03, 95% CI: 0.93–1.13).

**Conclusions:**

No adverse effects of cervical procedures on subsequent rates of pregnancy were observed in this cohort with up to twelve years of follow-up time.

## INTRODUCTION

Cervical cancer screening programs involving Papanicolaou (Pap) testing, and recently human papillomavirus (HPV) testing, have resulted in a marked decrease in invasive cervical cancer incidence worldwide.[1] These screening tests help identify early precancerous changes in the cervix or cervical intraepithelial neoplasia (CIN), which are caused by certain high-risk types of HPV (such as types 16 and 18) and can be removed surgically. Commonly performed procedures include loop excision (large loop excision of the transformation zone (LLETZ) or loop electrosurgical excision (LEEP)), cold knife conization, laser conization, cryotherapy, and laser ablation. These procedures are effective for removing precancerous lesions of the cervix and preventing progression to invasive cervical cancer [2–5]; however, they can increase the risk of preterm delivery among women who become pregnant after treatment.[6,7] Limited research has been done to understand the relationship between these procedures and subsequent fertility.

Surgical procedures on the cervix may contribute to infertility through several possible mechanisms. Cervical stenosis may occur after these procedures, which presents an anatomical barrier to sperm and fertilization.[8] Cervical mucus production may decrease after treatment, which could impair sperm motility.[9] And, rarely, infection may result from surgery which could lead to obstruction and adhesions within the fallopian tubes.[10]

Although each of these mechanisms is plausible, and they may work in concert to lower fertility for women who have had cervical procedures, there is limited research examining the effects of such procedures on fertility. Published studies to date have been limited to case reports, observational studies with small sample sizes, and studies using self-reported data which may be influenced by selective recall bias and misclassification.[6]

We examined pregnancy rates in a cohort of women 14–53 years of age at Kaiser Permanente Northwest from 1998 through 2009 to see whether treatments for precancerous cervical lesions were associated with lower pregnancy rates.

## MATERIALS AND METHODS

### Retrospective Matched Cohort

Kaiser Permanente Northwest (KPNW) is an integrated health care delivery system serving about 470,000 individuals in northwest Oregon and southwest Washington. We identified all female KPNW members aged 14 to 53 years during the period 1998–2009. We assigned each woman a study start date corresponding to the date upon which she joined the health plan during the 12-year study period, January 1, 1998 for women who were already enrolled at the beginning of the study period, or the date of her 14^th^ birthday. We censored cohort members and assigned them a study end date when they turned 55 years of age, terminated their health plan coverage, or as of December 31, 2009, whichever date occurred first. For women who left and rejoined the health plan during this period, we included all their enrollment periods.

We excluded women who were not “at risk” of becoming pregnant due to a history of hysterectomy, oophorectomy, sterilization, or diagnosed genetic infertility. A list of diagnosis and procedure codes used to define these exclusion criteria is available by request from the authors.

### Identification of Exposed Women

After applying the criteria described above, we identified women who underwent a cervical procedure during the study period using procedure codes. Excisional procedures included loop excision (LEEP or LLETZ) and conization (cold knife, laser, or loop electrode). Ablative procedures included laser ablation, cryosurgery, and cautery (electro or thermal).We assigned each of these women an index date, which was the first date of exposure to any of the qualifying treatment procedures and required her to have been enrolled in the health plan on the index date and for at least six continuous months leading up to the index date. We calculated age at the time of exposure for each woman and stratified the women by year of index date and five-year age categories. These categories were used for frequency matching with the two control groups described below.

### Comparison Group Exposed to Cervical Diagnostic Procedures Only

After identifying exposed women, we identified a comparison group of women who were exposed to diagnostic cervical procedures, such as a colposcopy and/or biopsy, during the period 1998–2009 but had not been exposed to cervical treatment procedures. We assigned each woman an index date, which was the first date of exposure to any of the qualifying diagnostic procedures; we required her to have been enrolled in the health plan on the index date and for at least six continuous months leading up to the index date.

### Comparison Group Unexposed to Any Cervical Procedures

For each woman who met the study inclusion criteria and had not been exposed to any of the diagnostic or treatment cervical procedures of interest during the period 1998–2009, we randomly assigned an index year during which she had been enrolled for at least one month. We calculated age in years for each woman on July 1 of her index year. We then stratified all the women by the assigned index year and five-year age categories.

We frequency-matched unexposed women to women exposed to cervical treatment within index year and five-year age category strata. Within each year-age stratum, we randomly assigned the index dates of the exposed women to the unexposed women as pseudo-index dates. We excluded unexposed women who had not been enrolled in the health plan on their assigned index date and for at least six continuous months leading up to this index date. Our final matching ratio of exposed women to unexposed women was 1:20.

### Data Collection

We collected information about health plan enrollment history, diagnoses, medical procedures, pharmacy dispensings, vital signs, and demographic descriptors from KPNW electronic medical records. The following list of covariates was selected for our analyses: age at index date, race/ethnicity, body mass index (BMI), cigarette smoking status, immunocompromised status, history of sexually transmitted infections (STIs) other than HPV, infertility diagnoses, testing and treatment, contraceptive use, obstetric history, length of prior health plan enrollment, and frequency of health care utilization. We created analytic variables for race, ethnicity and BMI by imputing values for these variables where they were missing. The BMI missing values were first categorized by age group and then assigned the mean BMI value of that specific age group. Most missing values for race/ethnicity were replaced by geo-coded race/ethnicity information from the U.S. 2000 Census. The values that were still missing after utilizing the geo-coded information were replaced by the modal value which was “white”.

We collected all available contraceptive use information for the study cohort members from the KPNW pharmacy, procedure, and diagnosis databases, and used this information to create a time-dependent variable representing time exposed and not exposed to a contraceptive method during the study period. We included oral contraceptives, injectable hormonal methods, implantable hormonal methods, and intrauterine devices (IUDs), but did not capture data on emergency contraceptive use, barrier methods, male partner sterilization, or abstinence.

We used a validated algorithm developed by KPNW researchers to identify a woman’s first pregnancy after the index date.[11] We included pregnancies of all outcome types, not just those ending in live births. The algorithm has good agreement with manual chart review for live births (100%), spontaneous abortions (93%), therapeutic abortions (96%) and stillbirths (88%).

### Propensity Score Trimming

We developed propensity scores for the likelihood of exposure to cervical surgical treatment. We computed separate propensity score models for the two comparison groups. All the variables in the propensity score were measured for the time period before the index date. Variables evaluated in propensity score model building included: age, race, ethnicity BMI, cigarette smoking status, immunocompromised status, STI history, obstetric history, diagnosis of infertility, length of prior health plan enrollment, and frequency of health care utilization. We then excluded women from the treatment and two comparison groups whose propensity scores did not overlap with each other.

### Analysis

We compared the women exposed to cervical treatment procedures to the women exposed to diagnostic procedures only and to the matched unexposed women using chi square and t-tests as appropriate. We also described contraceptive use and infertility diagnoses, testing, and treatment among the exposed women and two comparison groups during the study period.

We used Cox proportional hazards regression modeling to calculate the rate of pregnancy among exposed women compared to the rates in each of the two comparison groups. Calculation of person-time included days between the index date and the start date of the first pregnancy episode. Person-time was censored as described above at the end of the study period, health plan disenrollment, or a woman’s 55^th^ birthday. We plotted the Nelson-Aalen cumulative hazard distribution for all three study groups, and calculated hazard ratios stratified by treatment procedure type (excisional vs. ablative).

We conducted a formal assessment of potential confounders identifying variables that were statistically associated with both pregnancy and treatment and changed the estimate of the treatment effect by 10% or more when included in a bivariate regression model. Contraceptive use was included in the Cox models as a time-dependent covariate. All analyses were conducted using SAS Version 9.2 (SAS Institute, Cary, NC).

### Ethics Statement

The study protocol was reviewed and approved by the Kaiser Permanente Northwest Institutional Review Board (IRB). The study was conducted with a waiver of informed consent issued by the IRB. As part of their membership agreement with KPNW, health plan members sign a statement acknowledging that their medical records information may be used for research purposes; members who opted out of research at the time of health plan enrollment were excluded.

## RESULTS

**Fig 1:**
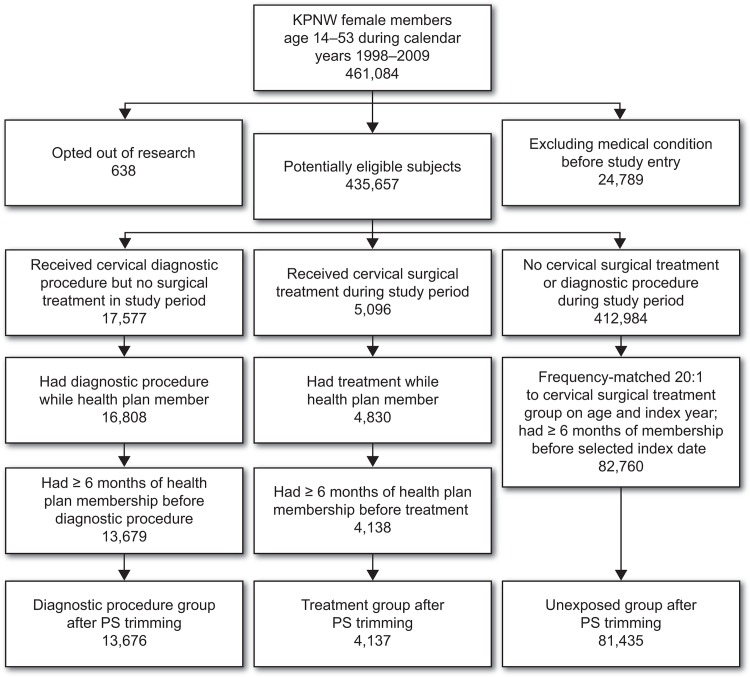
Inclusion and Exclusion Criteria to Establish a Cohort of Women Exposed to Cervical Treatment or Diagnostic Procedures and a Matched Sample of Unexposed Women, Kaiser Permanente Northwest, 1998–2009.

A total of 461,084 female KPNW health plan members 14–53 years of age were identified for the years 1998–2009 (Fig. 1). Of these, we excluded 638 women who opted out of participation in research studies and 24,789 with a previous hysterectomy or other excluding medical condition, leaving 435,657 potentially-eligible cohort members. After applying study eligibility criteria and propensity score trimming, we identified 4,137 women who received a cervical treatment procedure during the study period (exposed group), and 13,676 women who received a colposcopy and/or biopsy only during the study period (diagnostic procedure comparison group). Applying the 1:20 frequency matching protocol described above, we selected a comparison group of 81,435 women unexposed to either cervical treatment or diagnostic procedures.

The treatment and unexposed groups were similar in terms of age due to matching, but the diagnostic procedure group was slightly older than the treatment group (Table 1). Race, ethnicity, and immunocompromised status were consistent across the three groups. Several factors, including infertility, obstetric history, and STI diagnoses, were more common among the treatment group compared to the unexposed group; the distribution of these factors in the diagnostic procedure group was more closely aligned with the treatment group distribution.

**Table 1:** Characteristics of Women Exposed or Unexposed to Cervical Treatment or Diagnostic Procedures.

	Treatment (n = 4,127)	Diagnostic (n = 13,676)	p-value	Unexposed (n = 81,435)	p-value
	mean (s.d.)	mean (s.d.)		mean (s.d.)	
Age at Index Date (years)	32.15 (9.64)	34.45 (11.36)	<0.0001	31.53 (9.97)	<0.0001
Body Mass Index	26.98 (6.20)	27.22 (6.46)	0.03	27.45 (6.10)	<0.0001
	n (%)	n (%)		n (%)	
Age at Index Date (years)					
<20	286 (7.0)	1,313 (9.6)	<0.0001	7,448 (9.2)	<0.0001
20–29	1,613 (39.0)	4,173 (30.5)		31,674 (38.9)	
30–39	1,226 (29.7)	2,863 (20.9)		23,777 (29.2)	
≥40	1,002 (24.3)	5,327 (38.9)		18,536 (22.8)	
Race					
White	3,615 (87.6)	11,738 (85.8)	0.004	71,245 (87.5)	0.84
Non-White	512 (12.4)	1,938 (14.2)		10,190 (12.5)	
Ethnicity					
Hispanic	172 (4.2)	663 (4.9)	0.07	3,043 (3.7)	0.16
Non-Hispanic/Unknown	3,955 (95.8)	13,013 (95.1)		78,392 (96.3)	
Infertility^a^					
Yes	335 (8.1)	1,763 (12.9)	<0.0001	5,105 (6.3)	<0.0001
No	3,792 (91.9)	11,913 (87.1)		76,330 (93.7)	
Immunocompromised					
Yes	38 (0.9)	103 (0.8)	0.29	345 (0.4)	<0.0001
No	4,089 (99.1)	13,573 (99.2)		81,090 (99.6)	
Prior Obstetric History^b^					
Yes	1,121 (27.2)	3,356 (24.5)	0.0007	17,826 (21.9)	<0.0001
No	3,006 (72.8)	10,320 (75.5)		63,609 (78.1)	
Contraceptive Use					
Yes	2,134 (51.7)	6,175 (45.1)	<0.0001	25,204 (31.0)	<0.0001
No	1,993 (48.3)	7,501 (54.9)		56,231 (69.0)	
Sexually Transmitted Infection					
Yes	468 (11.3)	1,183 (8.7)	<0.0001	1,637 (2.0)	<0.0001
No	3,659 (88.7)	12,493 (91.3)		79,798 (98.0)	
Smoking					
Yes	1,656 (40.1)	4,204 (30.7)	<0.0001	16,691 (20.5)	<0.0001
No	2,471 (59.9)	9,472 (69.3)		64,744 (79.5)	
Health Plan Enrollment					
<15 months	833 (20.2)	2,585 (18.9)	<0.0001	20,694 (25.4)	<0.0001
15–36 months	1,161 (28.1)	3,245 (23.7)		24,267 (29.8)	
37–72 months	836 (20.3)	2,630 (19.2)		15,364 (18.9)	
≥72 months	1,297 (31.4)	5,216 (38.1)		21,110 (25.9)	
Health Care Visits per Year					
<2	197 (4.8)	1,048 (7.7)	<0.0001	17,414 (21.4)	<0.0001
3–4	1,111 (26.9)	4,854 (35.5)		27,564 (33.8)	
5–7	1,093 (26.5)	3,323 (24.3)		15,513 (19.0)	
≥8	1,726 (41.8)	4,451 (32.5)		20,944 (25.7)	

^a^ women with an infertility diagnosis, fertility testing, or fertility treatment

^b^ obstetric history defined as at least one pregnancy documented in the electronic medical record prior to the index date

Smoking was more common among the treatment group (40%) compared to the unexposed group (21%) and the diagnostic procedure group (31%). Length of prior enrollment and mean number of medical visits per year were modestly higher in the treatment group compared to the unexposed; however, the diagnostic procedure group had the largest proportion of women with >36 months prior membership in the health plan (57% diagnostic vs. 52% treatment vs. 45% unexposed).

Fifty-two percent of the treatment group used contraceptives at some time during the study period, compared to 31% of the unexposed group (p<.0001) and 45% of the diagnostic procedure group (p<.0001). In all groups, oral contraceptives were the most commonly-used method, followed by injectables, IUDs, and implants (data not shown).

We observed 570 pregnancies (14%) following surgical treatment (index date) in exposed women, 7,436 pregnancies (9%) in unexposed women after their index date, and 1,533 pregnancies (11%) following colposcopy/biopsy in the diagnostic procedure group. Compared to unexposed women, pregnancies were more common in the treatment group (hazard ratio (HR) = 1.35, 95% confidence interval (CI): 1.24–1.47) (Table 2). Similarly, the rate of pregnancies in the treatment group was higher than the rate in the diagnostic procedure comparison group (HR = 1.35, 95% CI: 1.22–1.48). Age and contraceptive use emerged as important confounders in both comparison groups in these models; pregnancy rates decreased with increasing age and during periods of contraceptive use. Infertility was also a confounder in the treatment vs. unexposed models. After adjusting for these confounders, we continued to observe a higher rate of pregnancies in the treatment group compared to unexposed women (HR = 1.42, 95% CI: 1.30–1.55), but there was no difference in pregnancy rates between the treatment group and the diagnostic procedure group (HR = 1.03, 95% CI: 0.93–1.13).

**Table 2:** Pregnancy among Women Exposed or Unexposed to Cervical Treatment orDiagnostic Procedures.

	Crude HR (95% CI)	Adjusted ^a^ HR (95% CI)
Cervical Treatment Procedure vs. Unexposed	1.35 (1.24–1.47)	1.42 (1.30–1.55)
Contraceptive Use (time-dependent)		0.28 (0.26–0.30)
Age at Index Date		
<20 years		1.0 (ref)
20–29 years		2.81 (2.60–3.04)
30–39 years		1.03 (0.95–1.13)
40+ years		0.04 (0.03–0.05)
Infertility Diagnosis, Treatment, or Testing		0.85 (0.77–0.93)
Cervical Treatment Procedure vs. Diagnostic Procedure	1.35 (1.22–1.48)	1.03 (0.93–1.13)
Contraceptive Use (time-dependent)		0.24 (0.22–0.28)
Age at Index Date		
<20 years		1.0 (ref)
20–29 years		0.88 (0.79–0.99)
30–39 years		0.31 (0.28–0.36)
40+ years		0.01 (0.01–0.01)

HR = hazard ratio;

CI = confidence interval

^a^ adjusted for all covariates listed

**Fig 2:**
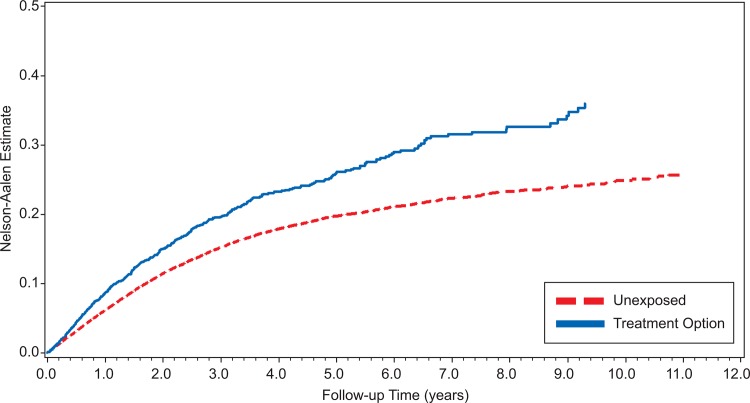
Time to Pregnancy among Women Exposed or Unexposed to Cervical Treatment Procedures over 12 Years of Follow-Up.

**Fig 3:**
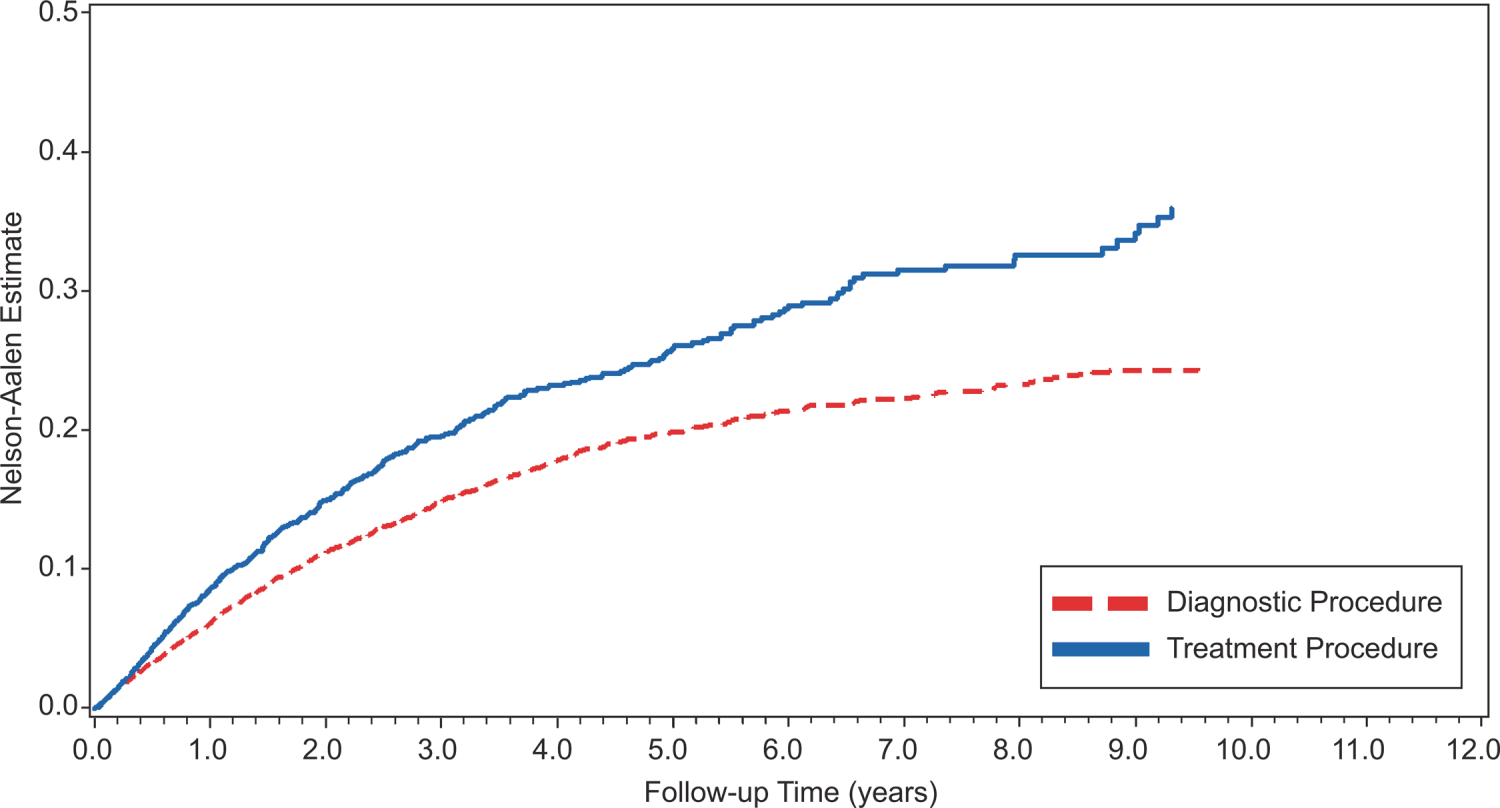
Time to Pregnancy among Women Exposed to Cervical Treatment Procedures or Diagnostic Procedures over 12 Years of Follow-Up.

Figs. 2 and 3 show Nelson-Aalen plots of the cumulative hazard in all three study groups. Across all years of follow up, women in the treatment group were more likely to become pregnant than women in either of the two comparison groups.

Nine hundred seventy-six women were exposed to ablative treatment procedures and 3,151 were exposed to excisional procedures. We observed similar adjusted hazard ratios for ablative procedures (HR = 1.40, 95% CI: 1.21–1.63) compared to excisional procedures (HR = 1.42, 95% CI: 1.29–1.58) using the unexposed comparison group. Similarly, there was little difference in adjusted hazard ratios for ablative (HR = 0.95, 95% CI: 0.81–1.11) and excisional procedures (HR = 1.07, 95% CI: 0.95–1.19) compared to women exposed to diagnostic procedures.

## DISCUSSION

In a cohort of over 435,000 women with up to 12 years of follow-up time, we did not observe any adverse effects of cervical treatment procedures on subsequent pregnancy rates. In fact, we observed increased pregnancy rates in women who underwent these procedures compared to unexposed women and women who received diagnostic procedures only. Several characteristics we measured suggest that the women in the treatment group may have been more sexually active than women in the comparison groups, but one major limitation of this study is that we were not able to assess sexual activity directly nor were we able to evaluate intention to become pregnant.

There are few published studies on this topic with which to compare our findings.[6] Four previous studies with control groups reported on total number of pregnancies, ability to conceive within a specified period, and time to pregnancy.[12–15] None of these studies suggest an adverse effect of cervical procedures on these outcomes. Consistent with our findings, one study found an increased probability of pregnancy in the treatment group compared to a matched unexposed group.[14] These prior studies were very heterogeneous in design, included small sample sizes, and may have been influenced by selection bias and misclassification because they primarily relied on survey data and fertility clinic records. Our study overcomes some of these limitations by examining a large, population-based cohort with outcomes and exposures derived from electronic medical records data rather than self-report. An additional strength of our study is that we were able to capture data on a number of important covariates, including contraceptive use, from our comprehensive databases.

Our findings that certain characteristics and behaviors, such as smoking and STI diagnoses, are more common among women exposed to these procedures is consistent with many other published studies.[12,16] To better control for these unbalanced covariates, we included a comparison group of women who underwent diagnostic procedures thinking that these women would also have been exposed to HPV and likely had sexual behaviors and exposures more similar to the treatment procedures group. This diagnostic procedure comparison group did indeed more closely resemble the exposed group, but there are likely still some unmeasured confounders we were unable to capture and control for in our analyses. As noted above, we were not able to directly ascertain sexual activity and intention to conceive, nor were we able to capture data on barrier contraceptive methods or abstinence.

While we believe our finding of increased pregnancy rates among women undergoing cervical treatments most likely represents unmeasured confounding, there may be an underlying biologic relationship between pregnancy and progression of HPV infection to precancerous lesions and cervical cancer. Several studies have reported that multiparity is associated with an increased risk of cervical intraepithelial neoplasia, and several authors have speculated that hormonal and immunologic changes during pregnancy may facilitate HPV DNA integration and progression of infection.[17,18] Alternatively, there may be factors (such as host immune responses) that both make successful pregnancy more likely and facilitate HPV integration and progression. Women undergoing cervical treatment or diagnostic procedures in our study were more likely to have been pregnant prior to their index date than unexposed women, but obstetric history did not emerge as an important confounder in our analysis. Additional research is needed to better understand possible underlying pathways between HPV infection, progression, and fertility.

Cervical cancer screening and early detection and surgical treatment of precancerous lesions have led to a decrease in cervical cancer incidence. Use of HPV vaccines should also lead to further declines in cervical cancer incidence in the future. We found no evidence to suggest that cervical treatments have a negative effect on the ability to become pregnant in the early years following treatment and in up to twelve years following treatment; however, these procedures have been associated with an increased risk of pregnancy outcomes such as preterm delivery.[6,7] Although there are still gaps in our understanding of how these treatments may influence fertility, these results should be reassuring to clinicians and to women undergoing these procedures during their childbearing years.

## References

[1] International Agency for Research on Cancer (2005) Cervix Cancer Screening In IARC Handbook of Cancer Prevention, Volume 10 Lyon: International Agency for Research on Cancer.

[2] Soutter WP, de Barros Lopes A, Fletcher A, Monaghan JM, Duncan ID, et al (1997) Invasive cervical cancer after conservative therapy for cervical intraepithelial neoplasia. Lancet 349:978–980. 9100623 10.1016/s0140-6736(96)08295-5

[3] Chew GK, Jandial L, Paraskevaidis E, Kitchener HC (1999) Pattern of CIN recurrence following laser ablation treatment: long-term follow-up. Int J Gynecol Cancer 9:487–490. 11240816 10.1046/j.1525-1438.1999.99066.x

[4] Martin-Hirsch P, Paraskevaidis E, Kitchener H (2000) Surgery for cervical intraepithelial neoplasia. Cochrane Database Syst Rev 2:CD001318 10796771 10.1002/14651858.CD001318

[5] Nuovo J, Melnikow J, Willan AR, Chan BK (2000) Treatment outcomes for squamous intraepithelial lesions. Int J Gynaecol Obstet 68:25–33. 10687833 10.1016/s0020-7292(99)00162-9

[6] Kyrgiou M, Koliopoulos G, Martin-Hirsch P, Arbyn M, Prendiville W, et al (2006) Obstetric outcomes after conservative treatment for intraepithelial or early invasive cervical lesions: systematic review and meta-analysis. Lancet 367:489–498. 16473126 10.1016/S0140-6736(06)68181-6

[7] Arybn M, Kyrgiou M, Simoens C, Raifu AO, Koliopolous G, et al (2008) Perinatal mortality and other severe adverse pregnancy outcomes associated with treatment of cervical intraepithelial neoplasia: meta-analysis. BMJ 337:a1284 10.1136/bmj.a1284 18801868 PMC2544379

[8] McLaren J (1967) Conservative management of cervical precancer. J Obstet Gynaecol Br Commonwealth 74:487–492 6033269 10.1111/j.1471-0528.1967.tb03980.x

[9] Weed JC, Curry SL, Duncan ID, Parker RT, Creasman WT (1978) Fertility after cryosurgery of the cervix. Obstet Gynecol 52:245–246 683667

[10] Coppleson M, Atkinson KH, Dalrymple JC (1992) Cervical squamous and glandular intraepithelial neoplasia: clinical features and review of management In Coppleson M, Monaghan JM, Morrow CP Tattersall MHN, editors. Gynecologic Oncology. Edinburgh: Churchill and Livingston pp. 590–595.

[11] Hornbrook MC, Whitlock EP, Berg CJ, Callaghan WM, Bachman DJ, et al (2007) Development of an algorithm to identify pregnancy episodes in an integrated health care delivery system. Health Services Research 42:908–927. 17362224 10.1111/j.1475-6773.2006.00635.x PMC1955367

[12] Weber T, Obel EB (1979) Pregnancy complications following conization of the uterine cervix (II). Acta Obstet Gynecol Scand 58:347–351. 525267 10.3109/00016347909154594

[13] Bigrigg A, Haffenden DK, Sheehan AL, Codling BW, Read MD (1994) Efficacy and safety of large-loop excision of the transformation zone. Lancet 343:32–34. 7905048 10.1016/s0140-6736(94)90881-8

[14] Spitzer M, Herman J, Krumholz BA, Lesser M (1995) The fertility of women after cervical laser surgery. Obstet Gynecol 86:504–508. 7675369 10.1016/0029-7844(95)00237-l

[15] Turlington WT, Wright BD, Powell JL (1996) Impact of the loop electrosurgical excision procedure on future fertility. J Reprod Med 41:815–818. 8951130

[16] Hagen B, Skieldestad FE (1993) The outcome of pregnancy after laser conization of the cervix. Br J Obstet Gynaecol 100:717–720. 8399008 10.1111/j.1471-0528.1993.tb14261.x

[17] Jensen KE, Schmiedel S, Norrild B, Frederiksen K, Iftner T, et al (2013) Parity as a cofactor for high-grade cervical disease among women with persistent human papillomavirus infection: a 13-year follow-up. Br J Cancer 108:234–239. 10.1038/bjc.2012.513 23169283 PMC3553518

[18] Luhn P, Walker J, Schiffman M, Zuna RE, Dunn ST, et al (2013) The role of co-factors in the progression from human papillomavirus infection to cervical cancer. Gynecol Oncol 128:265–270. 10.1016/j.ygyno.2012.11.003 23146688 PMC4627848

